# The Global Epidemic of the SARS-CoV-2 Delta Variant, Key Spike Mutations and Immune Escape

**DOI:** 10.3389/fimmu.2021.751778

**Published:** 2021-11-30

**Authors:** Dandan Tian, Yanhong Sun, Jianming Zhou, Qing Ye

**Affiliations:** National Clinical Research Center for Child Health, National Children’s Regional Medical Center, The Children’s Hospital, Zhejiang University School of Medicine, Hangzhou, China

**Keywords:** COVID-19, SARS-CoV-2 variants, mutations, vaccine, immune escape

## Abstract

During the COVID-19 pandemic, SARS-CoV-2 variants have emerged and spread worldwide. The Delta (B.1.617.2) variant was first reported in India in October 2020 and was classified as a “variant of concern (VOC)” by the WHO on 11 May, 2021. Compared to the wild-type strain, several studies have shown that the Delta variant is more transmissible and has higher viral loads in infected samples. COVID-19 patients infected with the Delta variant have a higher risk of hospitalization, intensive care unit (ICU) admission, and mortality. The Delta variant is becoming the dominant strain in many countries around the world. This review summarizes and analyses the biological characteristics of key amino acid mutations, the epidemic characteristics, and the immune escape of the Delta variant. We hope to provide scientific reference for the monitoring and prevention measures of the SARS-CoV-2 Delta variant and the development strategy of a second-generation vaccine.

## 1 Introduction

Over the last two decades, SARS -CoV-2 has been the third coronavirus known to cause severe acute respiratory disease in humans, following SARS-CoV in 2003 and MERS-CoV in 2012 (1–3). Coronavirus disease 2019 (COVID-19) caused by severe acute respiratory syndrome coronavirus 2 (SARS-CoV-2) has a deleterious impact on health services and the global economy (4–6). As of 8 October 2021, COVID-19 has spread rapidly to more than 200 countries, and there have been 236,599,025 confirmed cases of COVID-19, including 4,831,486 deaths (www.who.int).

At the end of January 2020, the D614G mutation, which turns aspartic acid (Asp) into glycine (Gly) at site 614 of the spike protein, was first discovered in the UK and quickly became the dominant epidemic strain in the world, attracting widespread attention (7, 8). The established nomenclature systems for naming and tracking SARS-CoV-2 genetic lineages by Nextstrain, GISAID (https://www.gisaid.org/), and Pango are currently in use by scientists. The SARS-CoV-2 variants were classified as “variant of concern (VOCs)” and “Variant of Interest, VOI)” by the WHO. At present, Alpha B.1.1.7 (known as 20I/501Y.V1, VOC 202012/01) (9), Beta B.1.351 (known as 501Y.V2) (10), Gamma P.1 (known as 501Y.V3) (11) and Delta B.1.617.2 (known as 478K.V1) (12) are defined as “variants of concern (VOCs)” by the WHO. Several studies have indicated that the Delta variant has higher transmissibility (13–15) and immune evasion than the early original virus strain and the other three VOCs. COVID-19 patients infected with Delta have a higher risk of hospitalization, ICU admission, and mortality (16–18). The Delta is becoming a prominent global strain globally, which has brought new challenges to the prevention and control of the COVID-19 pandemic.

### 1.1 The Biological Characteristics of Key Amino Acid Mutations in the Spike Protein of the SARS-CoV-2 Delta Variant

SARS-CoV-2 invades host cells by binding the spike protein to angiotensin-converting enzyme-2 (ACE2) (19–21). The SARS‐CoV‐2 spike protein is cleaved by furin into the S1 subunit and S2 subunit. The S1 subunit consists of an N-terminal domain (NTD) and a receptor-binding domain (RBD) and is responsible for binding to the host-cell ACE2 receptor. In comparison, the S2 subunit includes the trimeric core of the protein and is responsible for membrane fusion. The spike protein is the dominant neutralization target of monoclonal antibodies (mAbs), convalescent plasma, and vaccines (22–24). Therefore, mutations in the S protein affect the transmissibility, pathogenicity, and immune escape of SARS-CoV-2 variants. The Delta variant has accumulated nine amino acid mutations (T19R, G142D, FR156⁃157del, R158G, L452R, T478K, D614G, P681R, D950N) in the spike protein (25).

#### 1.1.1 L452R

The L452R mutation is located in the receptor-binding motif (RBM) region in the RBD region, containing residues that bind to ACE2 (26–28). Analysis of the SARS-CoV-2 spike protein revealed that the L452 residue does not directly contact the ACE2 receptor (29). Instead, L452, together with F490 and L492, forms a hydrophobic patch on the surface of the spike RBD. The L452R mutation may cause structural changes in this region that stabilize the interaction between the spike protein and the host cell’s ACE2 receptor, leading to increasing infectivity (26, 30). Deng X et al. observed that the entry efficiency into host cells of stable pseudoviruses carrying the L452R mutation was 6.7-22.5-fold higher in 293T cells and 5.8-14.7-fold higher in human airway lung organoids (HAOs) compared to D614G alone (293T cells and HAOs can stably express ACE2) (26). These results indicated that L452R mutation could increase the binding affinity of the spike protein to the host-cell receptor ACE2.

Wilhelm A et al. (31) found that authentic SARS-CoV-2 variants harboring L452R had reduced susceptibility to convalescent and vaccine-elicited sera and mAbs. Compared to B.1, the neutralization activity of convalescent sera against Delta was reduced by 5.33-fold. The neutralization activity of sera elicited by the mRNA vaccine against Delta was 2-fold weaker than B.1. In contrast to Kappa, authentic SARS-CoV-2 variants harboring L452R have a substantial resistance against imdevimab and bamlanivimab. Even at high concentrations, imdevimab was not effective against Delta, indicating high resistance. However, neutralization of Delta was moderately reduced with the clinically approved combination of casirivimab/imdevimab (31). In addition, another pseudovirus simulation showed that the L452R mutation could enhance the immune escape ability of the virus against convalescent plasma (32) and monoclonal antibodies (SARS2-01, SARS2-02, LY-CoV555, SARS2-32, X593, P2B2F6) (33).

#### 1.1.2 T478K

Compared with the other two B.1.617 lineages (B.1.617.1 and B.1.617.3), Delta (B.1.617.2) does not have the E484Q mutation but has a unique T478K mutation (25). An in silico molecular dynamics study on the protein structure of spike has predicted that the T478K mutation, substituting a non‐charged amino acid (threonine) with a positive one (lysine), may significantly alter the electrostatic surface of the protein and increase steric hindrance of the spike protein. These factors could enhance the binding affinity of RBD to ACE2 and enhance the ability of the virus to invade the host cell (34). Similarly, *in vitro* cell culture studies have shown that the Delta variant carrying T478K is more likely to undergo secondary mutation in a low titer antibody environment, leading to the failure of host antibody immunization (34).

#### 1.1.3 P681R

Interestingly, the P681R mutation in the S protein of the B.1.617 lineage is unique and newly identified in VOCs. The P681R mutation is located at the furin cleavage site (FCS; residues RRAR positioned between 682-5), and the cleavage of this region is the key to host cell entry (35). Several analyses have found that the P681R mutation affects viral replication dynamics and potentially determines the B.1.617 variants (36–38). Pseudoviruses carrying the P681R mutation showed that this mutation significantly increased the level of the cleaved S2 subunit and the level of the cleaved S2 subunit of the D614G/P681R mutation was significantly higher than that of D614G alone. *In vitro*, cell culture experiments revealed that the size of floating syncytia in the D614G/P681R mutant-infected culture was significantly larger than that in the D614G mutant-infected cell culture (39). These data suggested that the P681R mutation facilitates furin-mediated cleavage of the SARS-CoV-2 S protein, accelerates viral fusion, and promotes cell-cell infection.

In addition, the neutralization analyses of pseudoviruses showed that three monoclonal antibodies against RBD had 1.5-fold (1.2 ~2.65) decreased neutralization activity by against pseudoviruses with the D614G/P681R mutation. The neutralizing activity assay using the 19 sera elicited by the BNT162b2 vaccine (two doses) showed that pseudoviruses carrying the D614G/P681R mutation are significantly resistant to the vaccine-induced NAbs compared to the D614G pseudoviruses (39). These results suggested that the P681R mutation generated resistance to some mAbs and sera elicited by mRNA vaccines.

Stefano Pascarella et al. (40) reported that the surface electrostatic potential (EP) of the RBD of the spike protein is markedly increased. This is particularly noticeable in the Delta variant, which shows multiple replacements from neutral or negatively charged amino acids to positively charged amino acids. The EP in the spike protein of the Delta variant includes the uncharged and hydrophobic residue of Leu452 changing to the positively charged residue Arg and the neutral residue Thr changing to the positively charged Lys at position 478. The positive electrostatic potential can favor the interaction between the B.1.617.2+ RBD and the negatively charged ACE2, increasing the binding affinity of RBD to ACE2 receptor, thus conferring a potential increase in the virus transmission.

**Figure 1:**
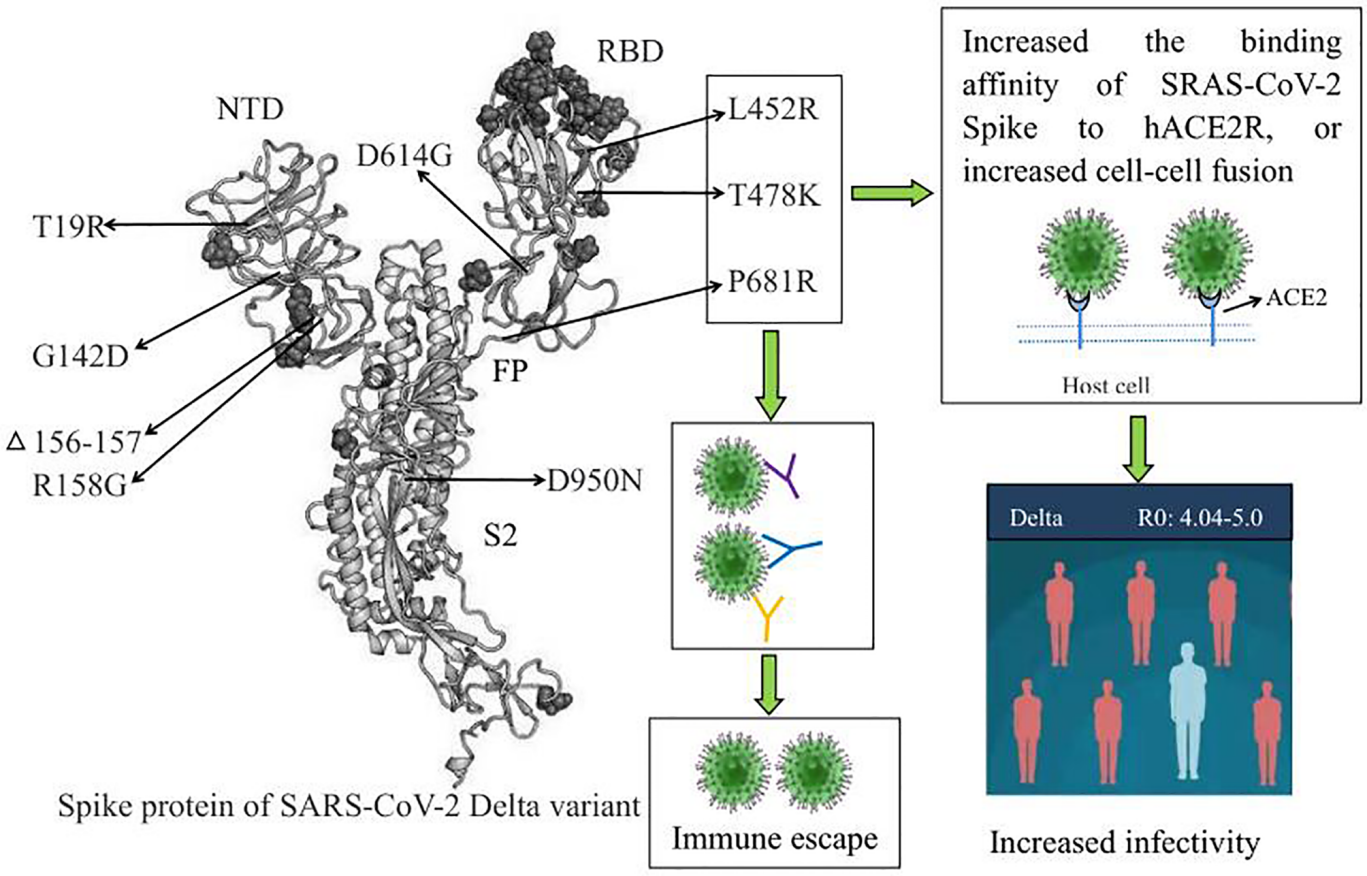
The biological characteristics of key amino acid mutations in the Spike protein of the SARS-CoV-2 Delta variant. L452R, T478K, and P681R are the three key amino acid mutations of the Delta variant, and these mutations increased transmissibility and generated immune escape of the Delta variant. RBD, receptor binding domain; NTD, N⁃terminal domain; ACE2, angiotensin-converting enzyme-2.

The above studies suggested that L452R, T478K, and P681R are the three key mutations of the SARS-CoV-2 Delta variant. These mutations increased transmissibility and generated immune escape of the Delta variant, as shown in **Figure 1**.

### 1.2 Delta Variant: More Transmissible, Shorter Incubation Period, Higher Viral Loads

Epidemiological investigation showed that the incubation period (the period of time from infection to illness onset) after infection with the Delta variant was 2-3 days, which was shorter than that of the wild-type strain (3-7 days). The basic reproductive number (R0, the infected person can transmit the pathogen to several other people) of the Delta variant (R0:4.04~5.0) was higher than that of the wild-type strain (R0:2.2~3.77). In addition, the generation time (GT, the interval between infection of the primary case and secondary cases) was 2.9 days (95% CI: 2.4–3.3), which was much shorter than the wild-type strain (2.9 *vs*. 5.7). It has been reported that the fifth generation of cases emerged just ten days after the first case was infected with the Delta variant (41–43).

One preprint from CDC Guangdong Province, China, had reported that the viral loads of patients infected with the Delta variant (n=62, Ct =24.00, IQR:19.00~29.00) were 1260-fold higher than those of the wild-type strain (n=63, Ct=34.31, IQR:31.00~36.00) when PCR was first used to detect SARS-CoV-2. Moreover, the number of patients infected with the Delta variant containing the viral loads > 6x105 copies/mL in oropharyngeal swabs was significantly higher than that of the wild-type strain when the viruses were first detected (80.65% *vs*. 19.05%) (44). In addition, the mean time of virus turning negative after the Delta variant infection was 13~15 days, which was much longer than that of wild-type strains of 7~9 d (45).

Moreover, epidemiological studies from Guangzhou also showed that patients infected with the Delta variant could spread in a short time even if individuals did not converse when sharing toilets or eat in the same space (46–48). Similarly, a follow-up case in Australia showed that a driver infected with the Delta variant was on the road, in a shopping mall, or in a cafe at the same time as three patients; virus transmission occurred despite a distance of only 10-60 cm (49). This suggests that the Delta variant may spread through aerosol in addition to the respiratory tract and close contact, leading to enhanced interpersonal transmission ability.

These data indicated that the Delta variant has higher transmissibility and a shorter incubation period. Patients infected with the Delta variant had higher viral loads (Ct value was less than 30).

### 1.3 The Delta Variant Is Becoming the Dominant Epidemic Strain in Many Countries Around the World

Delta (B.1.617.2) was first reported in India in October 2020 (12). Ram VS et al. reported that in India, the second wave started in March 2021, and they became the first country to report 400 000 cases per day by the end of April, and the emerged new Delta variant has played as a key infectious agent (50). The Delta variant has been linked to a resurgence of COVID-19 in Nepal and southeast Asia. Delta seems to be around 60% more transmissible than the already highly infectious Alpha variant (B.1.1.7) identified in the UK in late 2020 (51). From 20 to 27 May 2021, the total confirmed cases infected with the Delta variant increased from 3424 to 6959 in the UK (52, 53). Public Health England’s weekly coronavirus data on circulating variants showed 29,892 new cases of the Delta variant in the UK in the week (as of 9 June 2021), bringing the total number of cases of the Delta variant detected to 42,323 (54, 55).

According to nationwide sampling conducted by the genomics company Helix in San Mateo, California, Delta is rising fast, while Alpha fell from more than 70% of cases in late April to around 42% by mid-June 2021 (51). Since mid-June 2021, a sharp increase in COVID-19 cases has been observed in Israel, attributed to the Delta variant, which by mid-July 2021 constituted more than 95% of sequenced virus isolates in Israel (56, 57).

The first local infection of the Delta variant was identified in Guangzhou, Guangdong Province, China, on 21 May 2021 (44). From 21 May to 23 June 2021, the Delta variant caused epidemics in Guangzhou, Maoming, Foshan, Zhanjiang, and Shenzhen in Guangdong Province, China (46, 47). These results suggested that the Delta variant is becoming the dominant epidemic strain in many countries worldwide.

### 1.4 Delta Variant Has a Higher Risk of Hospital Admission, ICU Admission, and Mortality

Many clinical studies have reported that critical COVID-19 illness caused by infection with the wild-type strain includes acute respiratory distress syndrome; coagulopathies (58); septic shock; and multiple organ injuries, including liver injury (59), kidney injury (60), heart injury (61), and gastrointestinal symptoms (62). Preliminary data from Britain and Scotland showed that the hospitalization rate of patients infected with the Delta variant was 2-fold higher than that with Alpha (51). In Canada, a retrospective cohort study (63) showed that compared to non-VOC SARS-CoV-2 strains, the adjusted elevation in risk associated with N501Y-positive variants (B1.1.17, B.1.351 and P.1) was 59% (49-69%) for hospitalization, 105% (82-134%) for ICU admission, and 61% (40-87%) for death. Moreover, the adjusted risk of patients infected with the Delta variant was 120% (93-153%) for hospitalization, 287% (198-399%) for ICU admission, and 137% (50-230%) for death, which was significantly higher than N501Y-positive VOC variants, as shown in **Table 1**. In addition, the odds ratios (OR) for hospitalization, ICU admission, and death with Delta variant were as high as 2.20 (95% CI:1.93-2.53), 3.87 (95% CI:1.5-3.3), and 2.37 (95% CI:1.50-3.30), respectively (63). Similarly, the hazard ratios (HRs) of hospitalization for patients infected with the Delta variant was higher than that for patients infected with the wild-type strain in Scotland and in Singapore, with HRs:1.85 (95% CI:1.39-2.47) and HRs: 4.90 (95% CI:1.43-30.78), respectively (64). These studies showed that the risk of patients’ hospitalization, ICU admission, and mortality after Delta infection was higher than N501Y-positive VOC variants (B1.1.17, B.1.351, and P.1) and the wild-type strain, increasing the risk of severe COVID-19 disease.

**Table 1:** The epidemiological characteristics of the SARS-CoV-2 Delta variant.

WHO label	Delta
Pango lineage	B.1.617.2
Next strain	S:478K
GISAID clade	G/478K.V1
Amino acid mutations in the spike protein	T19R, G142D, FR156⁃157del, R158G, L452R, T478K, D614G, P681R, D950N (25)
Higher transmissibility	around 60% more transmissible than Alpha variant (51)
Higher risk of hospitalization, ICU admission, and mortality	the risk of patients infected with the Delta variant was 120% (93-153%) for hospitalization, 287% (198-399%) for ICU admission, and 137% (50-230%) for death, In Canada (63)
Immune escape	Resistance to partial mAbs, convalescent plasma, and partial vaccine
Shorter incubation period	2-3 days (Delta) *vs*. 3-7 days (Wild-type strain) (41–43)
Viral loads of patients infected with Delta when PCR first used to detect SARS-CoV-2	1260-fold higher than the wild-type strain (44)
The basic reproductive number: R0	4.04 ~ 5.0 (Delta) *vs*. 2.2~3.77 (Wild-type strain) (41–43)
The longer mean time of virus turning negative after the Delta variant infection	13~15 days (Delta) *vs*. 7-9 days (Wild-type strain) (45)

R0: The basic reproductive number.

### 1.5 Immune Escape From the Neutralization Activity of the Monoclonal Antibodies (mAbs) and Convalescent Plasma

It has been reported that the neutralization activity of 30% (6/20) of mAbs against the Delta variant was reduced more than 5-fold compared with that of the wild-type strain. In addition, the neutralization activity of 45% and 5% of convalescent plasma against Delta was reduced by approximately 3-10-fold and >10-fold, respectively (65, 66). Interestingly, the neutralization assay found that the neutralizing activity of convalescent plasma from individuals infected with the P.1 and B.1.351 variants against Delta was entirely lost, suggesting that individuals infected with B.1.351 and P.1 may be at risk of reinfection with the Delta variant (66).

### 1.6 Vaccine Efficacy Against Delta Variant

In Israel, two doses of the Pfizer vaccine (mRNA vaccine) can reduce symptomatic infections by 94%, related hospitalization by 87%, severe cases by 92%, and the risk of Delta infection by 79% (66, 67). In England, compared with patients infected with Delta who were not vaccinated, the risk of symptomatic infection caused by the Delta variant was decreased by 33%, and the hospitalization rate decreased by 75% three weeks after the first dose of the AstraZeneca or Pfizer vaccine. The effectiveness of the second dose of the AstraZeneca and Pfizer vaccines against Delta infection was increased by 60% and 88%, respectively. The hospitalization rate of patients infected with Delta who received two doses of the AstraZeneca and Pfizer vaccines was decreased by 92% and 96%, respectively (68–70).

The Delta variant can cause breakthrough infections in vaccinated populations in Guangzhou. The effectiveness evaluation of inactivated-virus vaccines-Sinovac/CoronaVac against the Delta variant during the epidemic in Guangdong showed that the efficacy of Sinovac/CoronaVac to prevent close contact infection was 69%, the efficacy of Sinovac/CoronaVac to prevent the development of symptomatic COVID-19 was 73%, and the efficacy of Sinovac/CoronaVac to prevent severe COVID-19 cases was 95% (45, 71).

All of these data revealed that, although the Delta variant presents partial immune escape, the mRNA vaccine (AstraZeneca and Pfizer vaccine) and inactivated-virus vaccines (Sinovac/CoronaVac) still have a protective effect against the Delta variant.

The severity of COVID-19 is mainly related to hosting factors, especially cellular immune responses in patients. Patients with mild COVID-19 and recovered patients with severe COVID-19 exhibit a normal humoral and a T cell-mediated immune response to effectively eliminate the virus (72). Vaccines induce neutralizing antibodies against the SARS-CoV-2 and induce a T cell response against the virus. Animal experiments have shown that a single intramuscular injection of the RNA-vaccine in mice elicited robust production of anti–SARS-CoV-2 S protein IgG antibody isotypes indicative of a type 1 T helper cell response. A prime/boost regimen induced potent T cell responses in mice, including antigen-specific responses in the lung and spleen (73). Another study showed that a single prime RNA- vaccine vaccination in mice led to robust neutralizing antibodies and produced a strong viral antigen-specific CD8+ T lymphocyte response (74). Moreover, several clinical studies have shown that individuals with prior infection can enhance T cell immunity against VOCs after one dose of mRNA vaccines (75, 76). mRNA vaccines generate antigen-specific T cells in a coordinated immune response, and vaccine-induced T cells resemble durable memory cells primed by infection (75, 76). These findings suggest that RNA vaccine-induced T cell responses are also involved in antiviral effects and neutralizing antibodies.

## 2 Conclusions

This review describes the biological characteristics of the L452R, T478K, and P681R mutations of the Delta variant spike protein. These mutations impact Delta variant biological behavior, including increased transmissibility and immune evasion. COVID-19 patients infected with Delta have a higher risk of hospitalization and ICU admission than patients infected with other VOCs (B1.1.17, B.1.351, and P.1) and wild-type strains. The Delta variant may be the most transmissible VOC and is becoming the main epidemic variant in many countries worldwide.

## Author Contributions

DT conceived and wrote the manuscript and prepared figures. YS and JZ contributed to the data collection and prepared the table. QY conceived and contributed to the modification and revision of the manuscript. All authors contributed to this article and approved the submitted versions.

## Conflict of Interest

The authors declare that the research was conducted in the absence of any commercial or financial relationships that could be construed as a potential conflict of interest.

## Publisher’s Note

All claims expressed in this article are solely those of the authors and do not necessarily represent those of their affiliated organizations, or those of the publisher, the editors and the reviewers. Any product that may be evaluated in this article, or claim that may be made by its manufacturer, is not guaranteed or endorsed by the publisher.
